# Frequency of FODMAP-rich products consumption and irritable bowel syndrome-like symptoms among amateur runners

**DOI:** 10.3389/fnut.2026.1888737

**Published:** 2026-07-28

**Authors:** Wiktoria Staśkiewicz-Bartecka, Paweł Lewandowski, Aleksandra Kołodziejczyk, Marek Kardas, Agata Kiciak

**Affiliations:** 1 Department of Food Technology and Quality Evaluation, Faculty of Public Health in Bytom, Medical University of Silesia, Katowice, Poland; 2 Department of Dietetics, Faculty of Public Health in Bytom, Medical University of Silesia, Katowice, Poland

**Keywords:** FODMAP, gastrointestinal symptoms, irritable bowel syndrome, physical activity, runners

## Abstract

**Background/objectives:**

Running may cause gastrointestinal symptoms resembling irritable bowel syndrome (IBS). One promising and widely studied tool to alleviate these symptoms is a diet restricting fermentable oligosaccharides, disaccharides, monosaccharides, and polyols (FODMAP). The aim of the study was to assess the relationship between the frequency of consumption of foods high in FODMAPs and the frequency of IBS-like symptoms among recreational runners.

**Materials and methods:**

The study included 153 recreational runners (81 women and 72 men) with a mean age of 37.8 ± 11 years. Most participants reported completing 3–4 training sessions per week (54.90%) and running 20–40 km per week (37.25%). Training intensity was not objectively assessed. Data were collected using the Computer-Assisted Web Interview method, a standardized questionnaire to assess the frequency of IBS-like symptoms, a proprietary questionnaire to assess behavioral and training factors, and food frequency questionnaire to assess the frequency of consumption of FODMAP-containing products.

**Results:**

Women scored significantly higher than men on the IBS symptom questionnaire (13.11 ± 4.82 vs. 10.71 ± 4.47; *p* = 0.002). Symptoms suggestive of IBS were observed more frequently among women than men (45.68% vs. 23.61%; *p* = 0.005). Gastrointestinal symptoms occurring during training were reported by 18.95% of all participants and were more frequent among men than women (26.39% vs. 12.35%), while post-training symptoms were reported by 12.42% of participants and were also more frequent among men than women (15.28% vs. 9.88%; overall *p* = 0.01). No statistically significant correlations were observed between the total FODMAP consumption frequency score and the IBS questionnaire score (*r* = 0.03; *p* = 0.76) or IBS questionnaire interpretation (*r* = -0.01; *p* = 0.89).

**Conclusion:**

No significant association was observed between the frequency of consumption of FODMAP-containing products and IBS-like symptoms in this sample of amateur runners. The observed differences in symptom severity and the characteristics of training activity suggest that individual factors and gastrointestinal adaptation may play a role in the experience of symptoms. These results provide a starting point for further research that could more precisely determine the mechanisms influencing the occurrence of IBS symptoms in physically active individuals.

## 1 Introduction

With the observed increase in public awareness of the relationship between insufficient physical activity and the increased risk of chronic diseases, significant interest in recreational sports such as running has been observed (1). This discipline is currently one of the most popular recreational sports (2–4). Endurance training is associated with numerous metabolic and cardiovascular benefits, improved body performance, and reduced risk of chronic diseases (3, 5, 6). However, long-distance running may also be associated with the occurrence of gastrointestinal (GI) symptoms (7). Gastroenterological complaints are frequently reported by runners and may result from temporary adaptive changes accompanying physical exercise, including redistribution of blood from the gastrointestinal tract, impaired intestinal motility, increased intestinal barrier permeability, and mechanical factors associated with running (8, 9). These changes result in symptoms that are usually acute and reversible, and may recur during training sessions or competitions (10). Because these symptoms partially overlap with those observed in irritable bowel syndrome (IBS), assessing them as IBS-like symptoms in runners is scientifically justified (8). Additionally, repeated exposure of the gastrointestinal tract to exercise stress and the specific dietary habits of physically active individuals may potentially contribute to more frequent reporting of gastrointestinal complaints in this population, justifying analysis of these symptoms. The recurrence of these symptoms may affect exercise tolerance, training comfort and sports performance, and consequently constitute a significant problem in the runners’ population (11, 12).

Irritable bowel syndrome (IBS) is one of the most common functional GI disorders (13). Its symptoms, such as bloating, diarrhea, and urgency to defecate, may overlap with gastrointestinal complaints reported during or after physical activity (11, 14–16). It should be emphasized that gastrointestinal symptoms occurring during or immediately after physical exercise are most often transient and do not constitute a diagnosis of a gastrointestinal disorder or irritable bowel syndrome. IBS is diagnosed based on clinical symptoms (17), and the occurrence and severity of symptoms can be modified through dietary interventions. In recent years, a diet low in fermentable oligo-, di-, and monosaccharides and polyols (FODMAPs) has attracted particular attention, leading to a reduction in symptom severity in some patients (15, 18, 19).

FODMAPs are a diverse group of carbohydrates and sugar alcohols characterized by a high degree of fermentation. Due to their limited absorption in the small intestine, they reach the large intestine, where they undergo bacterial fermentation, leading to increased gas production and increased osmotic pressure in the intestinal lumen (20). These mechanisms may exacerbate symptoms in individuals with visceral hypersensitivity. At the same time, FODMAP-rich foods are a natural and often desirable part of the diet (21–23). In the context of endurance sports, it has been argued that a high intake of FODMAPs, particularly around training, may increase the risk of GI symptoms during exercise (8). On the other hand, GI symptoms in athletes may result primarily from exercise-related factors (8, 9). These results emphasize the need for further research.

In Poland, where the present study was conducted, direct nationally representative estimates of total FODMAP intake are not available. However, national household food consumption data indicate regular consumption of food groups that may include FODMAP-containing products, such as bread and cereals, milk and dairy products, fruit, vegetables, and selected cereal-based products. These foods may contribute to exposure to fructans, lactose, excess fructose, galactooligosaccharides, and polyols, depending on the specific products consumed and portion sizes. A recent systematic review and meta-analysis estimated habitual total FODMAP intake in the general population at approximately 19.86 g/day, although this value should not be interpreted as a Poland-specific estimate. Therefore, in the present study, FODMAP exposure was assessed using a food-frequency approach focused on selected FODMAP-containing products rather than by estimating absolute FODMAP intake (24, 25).

The occurrence of GI symptoms among long-distance athletes is a popular topic among researchers. However, examining the impact of FODMAPs on their occurrence in this group is a relatively new area. Previous studies on the relationship between frequency of consumption of FODMAP-containing products and the occurrence of GI symptoms have focused primarily on advanced and elite athletes, often in small study groups. The diet of athletes, especially amateurs, may contain significant amounts of FODMAPs, which may exacerbate GI symptoms (8). The aim of this study was to assess the frequency of general IBS-like symptoms among amateur runners and to evaluate the relationship between the frequency of consumption of FODMAP-rich products and the severity of these symptoms. In addition, the study assessed whether reported gastrointestinal symptoms occurred during training, after training, or independently of physical activity.

## 2 Materials and methods

### 2.1 Procedure for the study

The study was conducted from November 2024 to May 2025. Participants were amateur runners who reported completing at least one training session per week at the time of the study. Data were collected during the running season, including both preparatory and competitive periods.

The study utilized a purposive sampling approach based on pre-defined inclusion criteria. These criteria were defined before the study began and used in the participant selection process.

The study was anonymous and participation was entirely voluntary. All participants provided informed consent to take part in the study. For participants under 18 years of age, informed consent was obtained from a parent or legal guardian, in addition to the participant’s assent. Information regarding the rules of participation was provided to respondents at the beginning of the survey. Approval for the study was obtained from the Bioethics Committee of the Medical University of Silesia in Katowice (decision no. BNW/NWN/0052/KB/229/23, dated 25 October 2023). Data was collected using the CAWI (Computer-Assisted Web Interview) method. The questionnaire was made available electronically via social media and distributed during selected running events. The Microsoft Office Forms platform was used for data collection. Only fully completed questionnaires were included in the analysis.

### 2.2 Participants

The study included 153 participants, including 81 (52.9%) women and 72 (47.1%) men, aged 15–67. The mean age of the participants was 37.8 ± 11 years.

Recruitment was conducted according to previously defined eligibility criteria. Inclusion criteria included: (1) informed consent to participate in the study, (2) being at least 15 years old, and (3) regular running training, understood as completing at least one training unit per week.

For the purposes of this study, amateur/recreational runners were operationally defined as individuals who reported regular running activity, defined as completing at least one running training session per week and declaring a weekly running distance within the predefined questionnaire categories. No minimum performance level, competition history, or running pace was required. The lowest weekly distance category available in the questionnaire was < 10 km/week; therefore, participants with low-volume but regular running activity were also eligible for inclusion.

Training volume was described using self-reported weekly training frequency and weekly running distance. Training intensity was not objectively assessed using running pace, heart-rate data, perceived exertion scales, or laboratory-based measures.

Exclusion criteria included: (1) being under 15 years old, (2) completing less than one training unit per week, and (3) incomplete or incorrectly completed questionnaire.

### 2.3 Survey tools

The study utilized a proprietary questionnaire created for the purposes of this study and a standardized Irritable Bowel Syndrome (IBS) questionnaire (26). The questionnaire was divided into four parts. The first part (1) involved obtaining informed consent from participants to participate in the study. The second part (2) was intended to collect metric data and information regarding training activity, including duration, volume, and frequency of training sessions, as well as dietary habits before and during training. This part also included information regarding a previous IBS diagnosis and adherence to a low-FODMAP diet. The third section (3) consisted of the IBS questionnaire, which assessed the presence and severity of IBS symptoms. This section also collected data on the circumstances of symptom onset. The final part (4) assessed the frequency of consumption of products relevant to the low-FODMAP diet and GI symptoms and included a proprietary dietary questionnaire based on a food frequency questionnaire (FFQ) (27).

#### 2.3.1 Body mass index (BMI)

The nutritional status of the participants was assessed using Body Mass Index (BMI), calculated on the basis of self-reported body weight and height. BMI values were classified according to World Health Organization criteria: underweight was defined as BMI < 18.5 kg/m^2^, normal weight as BMI 18.5–24.9 kg/m^2^, overweight as BMI ≥ 25.0 kg/m^2^, and obesity as BMI ≥ 30.0 kg/m^2^ (28).

#### 2.3.2 IBS questionnaire

A questionnaire developed by the World Gastroenterology Organization (WGO) was used to assess the presence and frequency of IBS-like symptoms. The questionnaire was developed in accordance with the Rome III criteria, which were the applicable IBS diagnostic criteria at the time of its development. It enables the assessment of common IBS-related symptoms, including abdominal pain, bloating, altered bowel habits, and changes in stool consistency.

The questionnaire has been previously validated in scientific studies, including a double-blind, controlled trial evaluating the effects of a symbiotic intervention on IBS symptoms, confirming its usefulness in assessing symptom occurrence and monitoring changes in symptom severity (26).

In the present study, the questionnaire was adapted to an electronic format and completed in a self-administered mode. In the present study, the questionnaire was used to assess the occurrence and frequency of IBS-like symptoms rather than to screen for or establish a clinical diagnosis of IBS. Respondents related their responses to the overall occurrence of symptoms over the past 3 months, regardless of physical activity. Additionally, to assess the potential relationship between general gastrointestinal symptoms and physical activity, two supplementary questions were used. These questions referred to all reported symptoms and addressed: (1) the circumstances of their occurrence (during training, after training, or regardless of physical activity) and (2) the subjective impact of physical activity on their severity (increases/does not increase). This approach allowed for the collection of consistent and comparable data while maintaining participant anonymity.

#### 2.3.3 Assessment of the frequency of consumption of FODMAP-containing products

To collect information on the frequency of consumption of FODMAP-containing foods, a custom-designed questionnaire based on the Food Frequency Questionnaire (FFQ) was used (27). The questionnaire included 46 food products, of which 8 were sources of fructose, 2 lactose, 4 mannitol, 8 sorbitol, 10 galactooligosaccharides, 17 fructans, and 12 products did not contain any FODMAP carbohydrate. Of these, 15 contained more than one FODMAP.

Food products were selected and assigned to specific groups according to the information contained in the Monash University application on FODMAP content in food (November 2024) (29). The frequency of consumption of each product was assessed using a seven-point scale:

- 0—I do not consume it,
- 1—I consume it 1–3 times a month,
- 2—I consume it once a week,
- 3—I consume it 2–3 times a week,
- 4—I consume it 4–6 times a week,
- 5—I consume it daily,
- 6—I consume it twice a day or more often.

Based on the assigned values corresponding to the frequency of consumption, a score was calculated for each FODMAP compound.

### 2.4 Statistical analysis

Statistical analyses were performed using LibreOffice Calc and Statistica v.13.3 (StatSoft Poland, Kraków, Poland). Quantitative variables were characterized using minimum (Min), maximum (Max), mean (X), and standard deviation (SD), while categorical variables were presented as numbers (n) and percentages (%).

Normality of distribution was assessed using the Shapiro–Wilk test. Differences between women and men in quantitative variables, including age, height, body mass, BMI, IBS questionnaire score, and FODMAP consumption frequency scores, were assessed using the Student’s *t*-test for independent samples when assumptions for parametric testing were met, or the Mann–Whitney U test when the distribution was non-normal.

For comparisons involving three or more independent categories, the Kruskal–Wallis ANOVA was used when the dependent variable was non-normally distributed or ordinal. This applied to analyses involving categories of training experience, weekly training frequency, weekly running distance, meal-to-training interval, and circumstances of gastrointestinal symptom occurrence.

The Chi-square test for independence was used to compare nominal variables between groups. These variables included BMI category, place of residence, education level, previous IBS diagnosis, low-FODMAP diet use, IBS-like symptom classification, training experience category, training frequency category, weekly running distance category, meal-to-training interval category, and circumstances of gastrointestinal symptom occurrence. Fisher’s exact test was used instead of the Chi-square test when expected cell counts were small.

Spearman’s rank correlation coefficient (R) was used to assess associations between ordinal or non-normally distributed variables, including training-related variables, FODMAP consumption frequency scores, IBS questionnaire score, and IBS questionnaire interpretation. The criterion for statistical significance was *p* < 0.05.

## 3 Results

### 3.1 Characteristics of the study group

The study group consisted of 153 individuals. 52.9% were women and 47.1% were men. Characteristics of the group are presented in Table 1.

**TABLE 1:** Group characteristics (*n* = 153).

Variable		Total (*n* = 153)	Women (*n* = 81)	Men (*n* = 72)	*p*-value
Age [years]		37.80 ± 11 (15–67)	37.54 ± 11.03 (16–57)	38.10 ± 11.04 (15–67)	0.95
Height [m]		1.73 ± 0.09 (1.52–1.95)	1.67 ± 0.06 (1.52–1.81)	1.79 ± 0.07 (1.60–1.95)	< 0.001***
Body mass [kg]		70.71 ± 12.59 (49–110)	64.22 ± 9.90 (49–98)	78.01 ± 11.27 (51–110)	< 0.001***
BMI [kg/m^2^]		23.67 ± 3.28 (17.65–36.81)	23.11 ± 3.30 (18.37–36.81)	24.30 ± 3.15 (17.65–33.08)	0.006*
Place of esidence	Rural area	30 (19.61%)	21 (25.93%)	9 (12.50%)	-
	Town < 20,000 inhabitants	21 (13.73%)	10 (12.35%)	11 (15.28%)	
	Town 20,000–100,000 inhabitants	41 (26.80%)	21 (25.93%)	20 (27.78%)	
	Town > 100,000 inhabitants	61 (39.87%)	29 (35.80%)	32 (44.44%)	
Education	Primary	7 (4.58%)	4 (4.94%)	3 (4.17%)	0.21
	Vocational	6 (3.92%)	1 (1.23%)	5 (6.94%)	
	Secondary	52 (33.99%)	25 (30.86%)	27 (37.50%)	
	Higher	88 (57.52%)	51 (62.96%)	37 (51.39%)	

Data are presented as mean ± standard deviation and range (minimum–maximum) for quantitative variables, or as number (n) and percentage (%) for categorical variables. Units are provided in the table headings. BMI, body mass index. Differences between women and men were assessed using the Student’s *t*-test or Mann–Whitney U test for quantitative variables and the Chi-square test or Fisher’s exact test for categorical variables, as appropriate.

*p < 0.05;

***p < 0.001.

BMI analysis revealed that the majority of study participants were within the healthy weight range (69.93%). BMI categories were interpreted according to WHO criteria, with underweight defined as BMI < 18.5 kg/m^2^. Underweight was reported in 1.96% of the study participants, overweight in 23.53%, and obesity in 4.58%. Underweight was reported in 1.23% of women and 2.78% of men. Overweight affected 16.05% of women and 31.94% of men, while obesity was reported in 3.7% of women and 5.56% of men, respectively. Gender differences in BMI interpretation were statistically significant (*p* = 0.006).

A previous medical diagnosis of IBS was self-reported by 15 participants (9.80%). This variable referred exclusively to a prior physician-confirmed diagnosis declared by the respondents and was not derived from the IBS symptom questionnaire.

Regarding the use of a low-FODMAP diet, the vast majority of respondents had never used it (94.12%), while 5.88% of participants reported using this type of diet. The differences between women and men in this regard were not statistically significant (*p* = 0.57).

In the study group, the largest percentage of participants had an amateur approach to running (*n* = 109; 71.24%). Approach to running was assessed based on participants’ self-assessment and reflected their subjective perception of their own engagement in running training. Statistical analysis revealed significant differences between women and men in terms of their declared approach to running (*p* = 0.003). Among women, amateur approach constituted 83.95% (*n* = 68), while among men, this percentage was 56.94% (*n* = 41). A professional approach was declared more often by men (*n* = 29; 40.28%) than by women (*n* = 13; 16.05%), while professional running was reported only by men (*n* = 2; 1.31%).

The training characteristics of the participants are presented in Table 2.

**TABLE 2:** Training characteristics of respondents by gender (*n* = 153).

Variable	Category	Total (*n* = 153)	Women (*n* = 81)	Men (*n* = 72)	*p*-value
Training experience	< 1 year n (%)	30(19.61)	22 (27.16)	8 (11.11)	0.0037**
	1–2 years n (%)	30 (19.61)	15 (18.52)	15 (20.83)	
	3–5 years n (%)	33 (21.57)	17 (20.99)	16 (22.22)	
	> 5 years n (%)	60(39.22)	27 (33.33)	33 (45.83)	
Training sessions per week	1–2 n (%)	41 (26.80)	30 (37.04)	11 (15.28)	0.0005***
	3–4 n (%)	84 (54.90)	42 (51.85)	42 (58.33)	
	5–6 n (%)	24 (15.69)	8 (9.88)	16 (22.22)	
	> 6 n (%)	4 (2.61)	1 (1.23)	3 (4.17)	
Weekly running distance	< 10 km n (%)	22(14.38)	15 (18.52	7 (9.72)	0.007**
	10–20 km n (%)	38 (24.84)	26 (32.10)	12 (16.67)	
	20–40 km n (%)	57 (37.25)	29 (35.80)	28 (38.89)	
	> 40 km n (%)	36(23.53)	10 (12.35)	26 (36.11)	

Data are presented as number (n) and percentage (%). Training experience is expressed in years, training frequency as the number of running training sessions per week, and weekly running distance as km/week. Differences between women and men were assessed using the Chi-square test or Fisher’s exact test, as appropriate.

**p < 0.01;

***p < 0.001.

Overall, Table 2 shows that most participants declared an amateur approach to running and most frequently reported completing 3–4 training sessions per week. Men more often reported a more performance-oriented approach to running, a higher number of weekly training sessions, and longer weekly running distances than women. These gender differences were statistically significant.

Analysis of the interval between meal and training showed that the most frequent interval reported was 60–120 min (43.79%), while the least frequent interval was < 30 min (5.23%). Gender differences in interval duration were statistically significant. Detailed data regarding the interval between meals and training are presented in Table 3.

**TABLE 3:** Meal-to-training interval data by gender (*n* = 153).

Group	Break between meal and training				*p*-value
	< 30 min n (%)	30–60 min n (%)	60–120 min n (%)	>120 min n (%)	
Women (*n* = 81)	7 (8.64)	24 (29.63)	36 (44.44)	14 (17.28)	0.007*
Men (*n* = 72)	1 (1.39)	16 (22.22)	31 (43.06)	24 (33.33)	
Total (*n* = 153)	8 (5.23)	40 (26.14)	67 (43.79)	38 (24.84)	

Data are presented as number (n) and percentage (%). The interval between meal intake and running training is expressed in min. Differences between women and men were assessed using the Chi-square test or Fisher’s exact test, as appropriate.

*p < 0.05.

### 3.2 IBS questionnaire assessment

Comparison of individual IBS-like symptoms reported in the IBS questionnaire showed significantly higher rates among women than men for abdominal pain (*p* = 0.02), hard stool (*p* = 0.01), difficulty with bowel movement or a feeling of incomplete evacuation (*p* = 0.03), bowel urgency (*p* = 0.04), and flatulence (*p* = 0.04). No statistically significant sex differences were observed for the remaining symptoms analyzed.

The mean score on the IBS symptom questionnaire among the respondents was 11.98 ± 4.80. Comparative analysis revealed statistically significant differences in scores between women and men (Table 4).

**TABLE 4:** IBS Questionnaire results, broken down by gender.

Group	World Gastroenterology Organization IBS symptom questionnaire [score]			
	X ± SD	Min	Max	*p*-value
Women (*n* = 81)	13.11 ± 4.82	3	23	0.002*
Men (*n* = 72)	10.71 ± 4.47	2	20	
Total (*n* = 153)	11.98 ± 4.80	2	23	

IBS-like symptoms were assessed using the World Gastroenterology Organization IBS symptom questionnaire, adapted to an electronic self-administered format. The questionnaire was used as a screening tool to assess the presence and severity of IBS-like symptoms and not to establish a clinical diagnosis of IBS. Data are presented as mean ± standard deviation and range for questionnaire scores, or as number (n) and percentage (%) for categorical variables. Differences between women and men were assessed using the Mann–Whitney U test for questionnaire scores and the Chi-square test or Fisher’s exact test for categorical variables, as appropriate.

*p < 0.05.

Based on the interpretation of the IBS symptom assessment tool, which assessed general IBS-like symptoms over the previous 3 months regardless of physical activity, 54 participants (35.29%) were classified as having symptoms suggestive of IBS. This result should be interpreted as the presence of IBS-like symptoms rather than as a clinical diagnosis of IBS. Symptoms occurring during training, after training, or independently of physical activity were assessed separately using supplementary questions.

In contrast, a previous medical diagnosis of IBS was self-reported by 15 participants (9.80%). Therefore, these two variables represent different constructs: prior physician-diagnosed IBS and current questionnaire-based IBS-like symptom burden. A score suggesting the presence of IBS-like symptoms was observed more frequently among women (*n* = 37; 45.68%) than among men (*n* = 17; 23.61%). This sex difference was statistically significant (*p* = 0.005).

Analysis of the circumstances of GI symptoms revealed that the highest percentage of respondents reported their presence regardless of training (*n* = 105; 68.63%). In this category, women (*n* = 63; 77.78%) were significantly more likely to report it than men (*n* = 42; 58.33%). Symptoms occurring during training were reported by 18.95% of all participants (*n* = 29), with this percentage being higher among men (*n* = 19; 26.39%) than women (*n* = 10; 12.35%). Post-training symptoms were reported by 12.42% of participants (*n* = 19), also more often by men (*n* = 11; 15.28%) than women (*n* = 8; 9.88%). Gender differences in the circumstances of GI symptoms were statistically significant (*p* = 0.01).

### 3.3 FODMAP intake characteristics

The mean score obtained on the frequency of consumption of FODMAP-containing products questionnaire was 65.45 ± 24.73 points on a 294-point scale. The observed range of scores ranged from 19 to 192 points. Differences between women and men were not statistically significant. Detailed results regarding the frequency of consumption of FODMAP-containing products among participants are presented in Table 5.

**TABLE 5:** Comparison of intake of selected FODMAP types among study participants.

Type FODMAP	Group	Consumption frequency score			
		X ± SD	Min	Max	*p*-value
Fructose	Women (*n* = 81)	15.47 ± 5.16	4	32	0.07
	Men (*n* = 72)	13.85 ± 5.86	1	34	
	Total (*n* = 153)	14.71 ± 5.54	1	34	
Lactose	Women (*n* = 81)	4.70 ± 3.46	0	12	0.65
	Men (*n* = 72)	4.83 ± 3.00	0	11	
	Total (*n* = 153)	4.76 ± 3.34	0	12	
Mannitol	Women (*n* = 81)	3.32 ± 1.82	0	13	0.63
	Men (*n* = 72)	3.36 ± 2.35	0	15	
	Total (*n* = 153)	3.34 ± 2.07	0	15	
Sorbitol	Women (*n* = 81)	7.58 ± 4.48	0	27	0.37
	Men (*n* = 72)	7.11 ± 5.30	0	34	
	Total (*n* = 153)	7.35 ± 4.87	0	34	
Galactooligosaccharides	Women (*n* = 81)	14.28 ± 5.87	5	39	0.054
	Men (*n* = 72)	12.65 ± 5.62	3	38	
	Total (*n* = 153)	13.52 ± 5.79	3	39	
Fructans	Women (*n* = 81)	22.11 ± 8.07	6	46	0.30
	Men (*n* = 72)	21.39 ± 9.97	7	63	
	Total (*n* = 153)	21.77 ± 8.99	6	63	

The frequency of consumption of FODMAP-containing products was assessed using a proprietary food frequency questionnaire based on FFQ methodology. Consumption frequency was scored on a seven-point scale from 0 to 6, where 0 indicated no consumption and 6 indicated consumption twice daily or more often. Data are presented as mean ± standard deviation and range. Differences between women and men were assessed using the Student’s *t*-test or Mann–Whitney U test, as appropriate.

### 3.4 Correlation between analyzed variables and IBS questionnaire scores

Spearman’s rank correlation analysis revealed no statistically significant correlations between any of the analyzed training variables, including the interval between a meal and training, and the obtained scores or interpretation of the IBS questionnaire results. Detailed results are presented in Table 6.

**TABLE 6:** Correlation assessment between training variables and IBS questionnaire scores (*n* = 153).

Variable	IBS score	IBS interpretation
Length of training experience	*r* = -0.05 (*p* = 0.54)	*r* = -0.005 (*p* = 0.95)
Approach to running	*r* = -0.06 (*p* = 0.43)	*r* = 0.04 (*p* = 0.57)
Number of training units per week	*r* = -0.06 (*p* = 0.42)	*r* = 0.02 (*p* = 0.81)
Weekly distance	*r* 0.05 (*p* = 0.53)	*r* = 0.07 (*p* = 0.39)
Meal-training interval	*r* = 0.10 (*p* = 0.22)	*r* = 0.06 (*p* = 0.39)

Correlations were assessed using Spearman’s rank correlation coefficient. R indicates Spearman’s correlation coefficient. IBS questionnaire score refers to the total score obtained using the IBS symptom questionnaire, while IBS questionnaire interpretation refers to the questionnaire-based classification of symptoms suggestive of IBS.

Assessment of the relationship between the frequency of consumption of FODMAP-containing products and IBS questionnaire scores revealed no statistically significant correlations. Detailed data regarding the correlations between individual FODMAP types and the obtained scores and interpretation of the IBS questionnaire are presented in Table 7.

**TABLE 7:** Assessment of correlations between the frequency of consumption of FODMAP-containing products and IBS questionnaire scores (*n* = 153).

FODMAP type	IBS score	IBS interpretation
Fructose	*r* = -0.02 (*p* = 0.77)	*r* = -0.06 (*p* = 0.44)
Lactose	*r* = 0.01 (*p* = 0.90)	*r* = -0.01 (*p* = 0.86)
Mannitol	*r* = 0.09 (*p* = 0.29)	*r* = 0.09 (*p* = 0.29)
Sorbitol	*r* = -0.08 (*p* = 0.34)	*r* = -0.10 (*p* = 0.21)
Galactooligosaccharides	*r* = 0.08 (*p* = 0.30)	*r* = 0.06 (*p* = 0.43)
Fructans	*r* = 0.08 (*p* = 0.30)	*r* = 0.05 (*p* = 0.56)
FODMAP total	*r* = 0.03 (*p* = 0.76)	*r* = -0.01 (*p* = 0.89)

Correlations were assessed using Spearman’s rank correlation coefficient. R indicates Spearman’s correlation coefficient. FODMAP consumption frequency scores were calculated from the proprietary food frequency questionnaire, based on the reported frequency of consumption of products containing specific FODMAP groups. IBS questionnaire score refers to the total score obtained using the IBS symptom questionnaire, while IBS questionnaire interpretation refers to the questionnaire-based classification of symptoms suggestive of IBS.

The analysis of the relationship between the frequency of consumption of various types of FODMAPs and the severity and circumstances of the occurrence of GI complaints due to physical activity did not show any statistically significant relationships in any of the analyzed groups.

## 4 Discussion

The primary hypothesis of this study was that a higher frequency of consumption of FODMAP-rich products would be associated with a higher frequency and severity of IBS-like symptoms among amateur runners. Overall, this hypothesis was not confirmed, as no statistically significant associations were observed between the frequency of consumption of FODMAP-containing products and IBS questionnaire scores or their interpretation. The main findings were that women reported a higher burden of general IBS-like symptoms than men, whereas men reported a higher training volume and more frequent gastrointestinal symptoms during or after training.

The IBS questionnaire results showed that women scored significantly higher than men, and the interpretation of the tool more frequently indicated the presence of symptoms suggestive of IBS. These observations are consistent with previous literature indicating that IBS is more common in women than in men across many populations (13, 30–32). The higher frequency of IBS-like symptoms among women may also be explained by the fact that these symptoms were reported regardless of training.

The study also showed statistically significant differences in the frequency of selected general symptoms associated with irritable bowel syndrome (IBS) in the last 3 months. Women were more likely than men to experience abdominal pain, hard stools, difficulty with defecation, a sensation of incomplete evacuation, urgency, and bloating. These symptoms are typical of constipation-predominant IBS and may suggest its higher prevalence among women. Previous studies indicate that these differences may be associated with hormonal factors and differences in pain perception, which supports the observations presented in this study (33).

A study by Killian et al. demonstrated a high frequency of consumption of FODMAP-containing products among endurance athletes, which may contribute to the occurrence of GI symptoms during exercise (34). Wiffin et al. (35) also confirmed that a short-term low-FODMAP dietary intervention resulted in a reduction in GI symptoms in physically active individuals, supporting the potential role of fermentable carbohydrates in the development of exercise-related symptoms.

In contrast to these findings, the present study did not demonstrate significant associations between the frequency of consumption of foods classified as sources of specific FODMAP groups and IBS questionnaire scores, and therefore did not support the primary research hypothesis that a higher frequency of consumption of FODMAP-containing foods would be associated with a higher prevalence of IBS-like symptoms among recreational runners.

Evidence from non-athletic IBS populations suggests that FODMAPs may contribute to gastrointestinal symptoms through poor absorption, osmotic activity, and rapid fermentation, leading to increased intestinal water content and gas production. These mechanisms may promote abdominal pain, bloating, flatulence, diarrhea, and altered bowel habits in susceptible individuals. Accordingly, previous studies and dietary intervention trials have shown that reducing FODMAP intake may improve global IBS symptoms, particularly bloating, abdominal pain, flatulence, and diarrhea (36–38).

In contrast to these findings, the present study did not demonstrate significant associations between the frequency of consumption of products classified as sources of specific FODMAP groups and IBS questionnaire scores. Therefore, the results did not support the primary research hypothesis that a higher frequency of consumption of FODMAP-containing products would be associated with a higher frequency of general IBS-like symptoms among recreational runners. One possible explanation for this finding may be gastrointestinal adaptation in individuals regularly exposed to training loads. Chronic exposure of the GI tract to exercise-related stress may promote the development of tolerance to specific foods (39, 40). This strategy, referred to as gut training, is widely used in both scientific and athletic contexts. Such adaptation may reduce the risk or severity of GI symptoms (7, 40), which in turn may explain the lack of a significant association between the frequency of consumption of FODMAP-containing products and symptom occurrence in the study population.

It is also worth noting that poor tolerance to FODMAPs is often observed in individuals with IBS (36–38), whereas the present study included a non-clinical sample of amateur runners and assessed IBS-like symptoms rather than physician-diagnosed IBS. The discrepancies between previous studies and the present findings may therefore be attributable primarily to methodological differences. In particular, previous studies have often focused on acute gastrointestinal symptoms during exercise or on the effects of dietary interventions, whereas this study assessed the relationship between the frequency of FODMAP-rich product consumption and general IBS-like symptoms over the previous 3 months. It is also important to consider that physical activity itself can influence gastrointestinal function. Moderate physical activity is associated with improved quality of life and reduced symptom severity in people with IBS, whereas intense and prolonged exercise can lead to symptom exacerbation. This indicates that the association between physical activity and IBS symptoms may depend on both exercise intensity and individual gastrointestinal susceptibility (41). Consequently, the lack of significant associations observed in this cross-sectional setting may be related both to the limited variability in the frequency of consumption of FODMAP-containing products and to adaptive mechanisms of the gastrointestinal tract in runners, rather than indicating a complete lack of relevance of these compounds for IBS-related symptoms.

The etiopathogenesis of IBS is complex and multifactorial. Chronic stress, previous infections, comorbidities, intestinal dysbiosis, as well as demographic and genetic factors are known to influence the occurrence and severity of IBS symptoms (42–45). This may suggest a relatively limited role of FODMAPs in the development of IBS symptoms, particularly among individuals who have not previously experienced gastrointestinal complaints. Furthermore, FODMAP-rich foods consumed outside the peri-exercise period may have a limited impact on the occurrence of GI symptoms during exercise. Moderate physical activity itself may contribute to the reduction of GI symptoms in individuals with IBS, which may also influence the symptom profile observed in the study group (46).

In addition to FODMAPs, other dietary factors may influence the occurrence of GI disturbances during exercise. These include high fiber and fat intake in pre-exercise meals, caffeine consumption (47), and inadequate hydration. These factors were not assessed in the present study, but they may significantly influence the occurrence of IBS-like symptoms, even in the context of a low frequency of consumption of FODMAP-containing products. Hydration is particularly important, as insufficient fluid intake may contribute to the occurrence of IBS symptoms, especially constipation-predominant IBS (48–50). Prolonged physical activity significantly increases fluid requirements, which may further influence the symptoms experienced by participants (8).

Another factor that may explain the lack of association between the frequency of consumption of FODMAP-containing products and IBS-like symptoms among runners is their lifestyle. Individuals who engage in regular physical activity may possess greater nutritional knowledge compared to sedentary individuals (51, 52). Furthermore, physical activity is often part of a broader set of health-promoting behaviors. Although most participants did not report following a low-FODMAP diet, their overall lifestyle habits, including sleep hygiene, stress management, and general dietary patterns, may indirectly influence the frequency of IBS-like symptoms in this population (53–55).

In the study group, statistically significant differences were found between the gender regarding the interval between a meal and training. Men were significantly more likely to begin physical activity more than 120 min after a meal compared to women, while women were more likely to engage in activity within less than 30 min and between 30 and 60 min after a meal compared to men. The study also revealed significant differences between genders in the occurrence of GI symptoms during and immediately after training, with men more likely to report GI symptoms both during and after exercise. However, the study did not demonstrate a significant correlation between the interval between a meal and training and the IBS questionnaire score or its interpretation. This may be related to the volume and composition of the pre-training meal. An easily digestible, small meal remains in the stomach for a shorter period of time compared to a heavy, large-volume meal (29, 30). However, the present study did not include a detailed analysis of pre-training meal characteristics. Furthermore, men demonstrated a higher training volume, which may constitute an additional factor contributing to GI disturbances around training, as the intensity and duration of exercise increase the risk of intestinal ischemia and digestive disorders (6, 10, 41).

This study has several limitations. First, the assessment of FODMAP-containing product consumption was based on self-reported frequency and did not include portion size or a quantitative estimation of FODMAP intake. Therefore, the results should be interpreted as reflecting the frequency of exposure to selected FODMAP-containing products rather than actual FODMAP intake. Second, all data were self-reported, which may be associated with recall bias and reporting bias. Third, the cross-sectional design does not allow causal relationships to be established. Fourth, the study did not assess the composition, portion size, or timing of pre-training meals in detail, although these factors may influence gastrointestinal symptoms during exercise. Additionally, due to the multifactorial etiology of irritable bowel syndrome, the study did not assess psychosocial factors, such as stress levels, anxiety, or sleep quality, which may influence the severity of IBS symptoms. Specific exclusion criteria regarding comorbidities, upper age limit, or adherence to a low-FODMAP diet were not included, as this study was designed to analyze a population of recreational runners in a real-world setting. However, these factors may influence the results and should be considered in the interpretation and future studies. Fifth, IBS-like symptoms were assessed using a questionnaire and were not confirmed by clinical diagnosis. Finally, the study sample consisted of amateur runners recruited through online channels and selected running events, which may limit the generalizability of the findings. A formal a priori power calculation was not conducted before data collection, and the sample size was determined by the feasibility of recruitment among amateur runners during the study period. A *post hoc* sensitivity analysis indicated that, assuming a two-tailed test, α = 0.05, statistical power of 80%, and the available sample size of *n* = 153, the study was powered to detect a correlation coefficient of approximately *r* = 0.225. Therefore, the study may have been underpowered to detect very small associations between the frequency of consumption of FODMAP-containing products and IBS-like symptoms. This should be considered when interpreting the absence of statistically significant correlations. Despite these limitations, the study has several strengths, including the relatively large sample size of amateur runners, the assessment of different categories of FODMAP-containing products, and the simultaneous analysis of dietary, gastrointestinal, and training-related variables.

In summary, the results of this study indicate that no significant association was found between the frequency of consumption of FODMAP-rich products and the frequency or severity of IBS-like symptoms in this population of amateur runners. The observed differences in symptom severity between women and men, as well as the characteristics of exercise activity, suggest that individual factors and gastrointestinal adaptation may play a role in the experience of symptoms, although their impact in the study sample remains unclear. These findings provide a basis for further research aimed at more precisely identifying the mechanisms underlying the occurrence of IBS-related symptoms in physically active individuals.

## Data Availability

The raw data supporting the conclusions of this article will be made available by the authors, without undue reservation.
